# Supplementary material for: Reproducing extracellular matrix adverse remodelling of non-ST myocardial infarction in a large animal model
Source: Nat Commun. 2023 Feb 22;14:995. doi: 10.1038/s41467-023-36350-1 (PMC9945840; doi:10.1038/s41467-023-36350-1)
Supplement: Supplementary file 1 — Supplementary Information [file 41467_2023_36350_MOESM1_ESM.pdf]

Supplementary Information

Supplementary Figures 2
Supplementary Figure 1 | Surgical procedure of NSTEMI induction in sheep 2
Supplementary Figure 2 | Comparison between STEMI and NSTEMI model 4
Supplementary Figure 3 | Resemblance of NSTEMI model to clinical cases 6
Supplementary Figure 4 | Functional and histological evaluation of the NSTEMI model 7
Supplementary Figure 5 | Transcriptomics in ischaemic core following NSTEMI 8
Supplementary Figure 6 | HIF1α Causal Network Analysis (IPA®) in ischaemic core post-NSTEMI 10
Supplementary Figure 7 | Functional annotation analysis on transcriptomics 12
Supplementary Figure 8 | Glycomics analysis of infarcted hearts following NSTEMI 13
Supplementary Figure 9 | HS sulfation pattern following NSTEMI 16
Abbreviations 16

Supplementary Figures

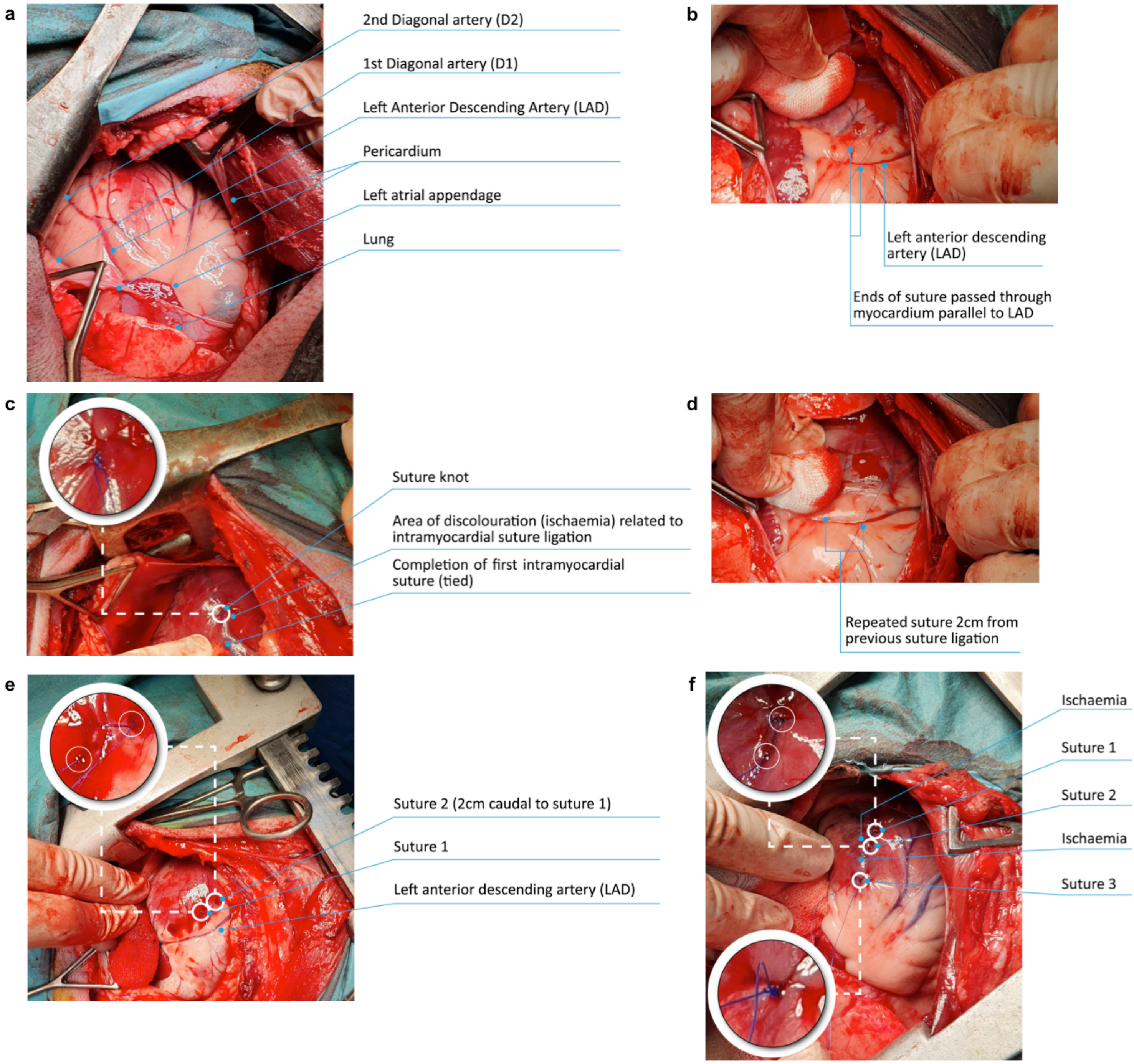

Supplementary Figure 1 | Surgical procedure of NSTEMI induction in sheep

a, Following thoracotomy and pericardiectomy, the heart is exposed to detect LAD and diagonal arteries. **b**, A 2/0 Prolene® suture is passed through the left ventricular myocardium parallel to the LAD. **c**, The first suture knot is completed and an initial area of discoloration appears in the proximity of the ligated ventricular portion. **d**,**e**, A second suture is performed approximately 2 cm apart from the first one, always parallel to LAD. **f**, Progressively, a third suture is performed and tied in the same direction towards the apex of the heart again 2 cm apart from the previous suture. The pale area of ischemia across the suture knots is evident.

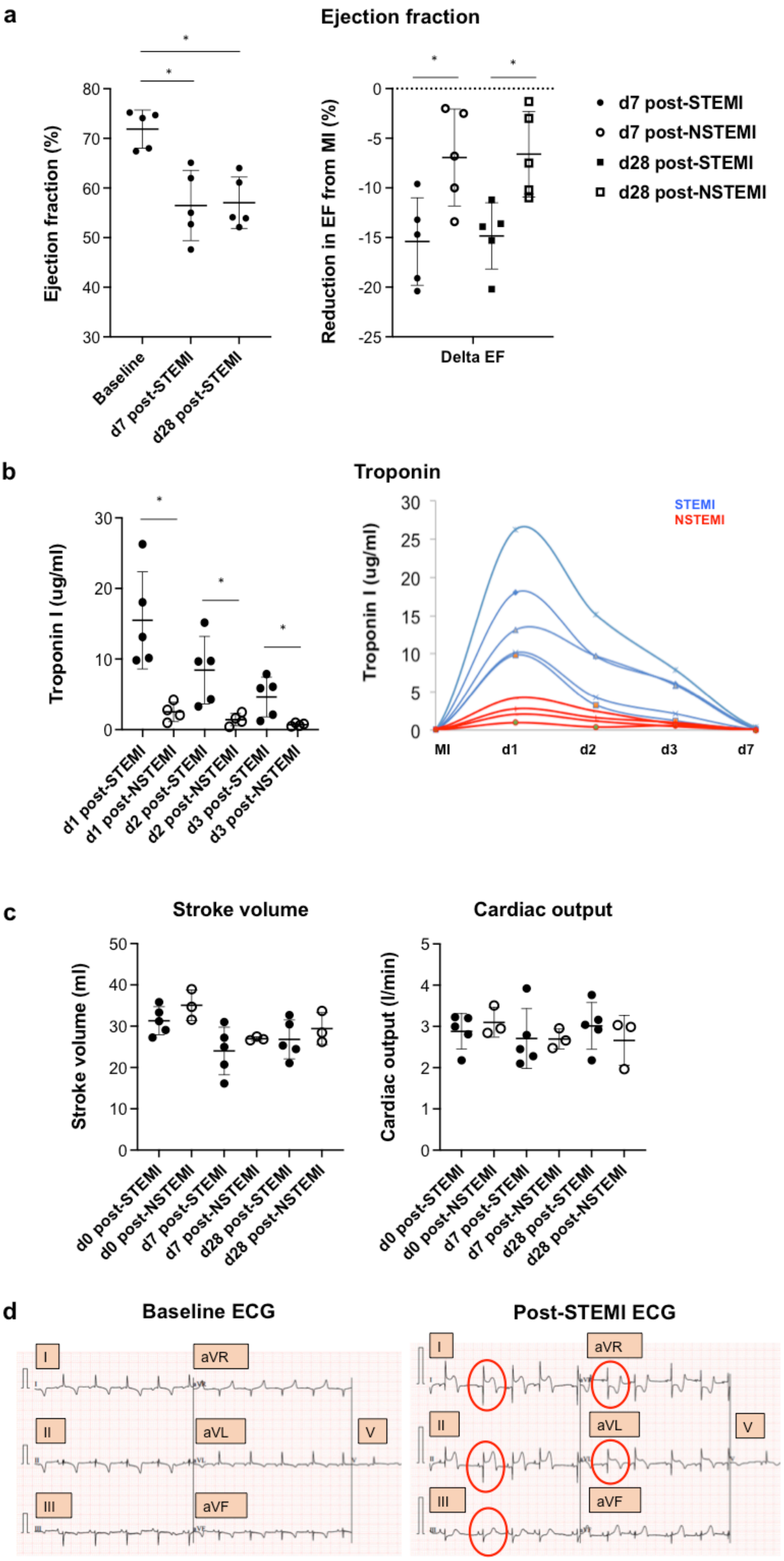
a
Ejection fraction
Ejection fraction (%)
Baseline
d7 post-STEMI
d28 post-STEMI
Reduction in EF from MI (%)
Delta EF
d7 post-STEMI
d7 post-NSTEMI
d28 post-STEMI
d28 post-NSTEMI
b
Troponin
Troponin I (ug/ml)
d1 post-STEMI
d1 post-NSTEMI
d2 post-STEMI
d2 post-NSTEMI
d3 post-STEMI
d3 post-NSTEMI
STEMI
NSTEMI
MI
d1
d2
d3
d7
c
Stroke volume
Stroke volume (ml)
Cardiac output
Cardiac output (l/min)
d0 post-STEMI
d0 post-NSTEMI
d7 post-STEMI
d7 post-NSTEMI
d28 post-STEMI
d28 post-NSTEMI
d
Baseline ECG
Post-STEMI ECG
I
aVR
II
aVL
V
III
aVF

Supplementary Figure 2 | Comparison between STEMI and NSTEMI model

a, Left, ejection fraction (EF) absolute values before ligation (baseline), 7 (d7) and 28 (d28) days after full-occlusion ligation. Right, comparison of the relative decrease in EF on d7 and d28 between STEMI and NSTEMI. *n=5* per group. Animals had the same age and weight. **b**, Left, mean troponin I level from d1 to d3 following either STEMI or NSTEMI induction. *n*=5 STEMI and *n*=4 NSTEMI animals. Right, individual troponin I level from ligation to d7 post-surgery in STEMI and NSTEMI. *n*=5 STEMI and *n*=4 NSTEMI animals. **c**, Haemodynamics of STEMI and NSTEMI in sheep measured as stroke volume (left) and cardiac output (right). *n*=5 STEMI and *n*=3 NSTEMI animals. **d**, Representative electrocardiogram (ECG) before STEMI-induction (left) and post-ligation (right). Marked ST elevation in leads I, II and aVL, reciprocal ST depression in lead aVR and milder ST depression in lead III are circled in red. *n*=5 animals. Kruskal-Wallis with Dunn's multiple comparisons test (*P*=0.018 for baseline vs. d7 and *P*=0.014 for baseline vs. d28) in (**a**, left) and (**c**), one-way ANOVA with Tukey's multiple comparisons test (*P*=0.029 for d7 post-STEMI vs. d7 post-NSTEMI, *P*=0.035 for d28 post-STEMI vs. d28 post-NSTEMI) in (**a**, right), two-tailed Mann-Whitney test (*P*=0.016 for d1 post-STEMI vs. d1 post-NSTEMI, *P*=0.016 for d2 post-STEMI vs. d2 post-NSTEMI, *P*=0.016 for d3 post-STEMI vs. d3 post-NSTEMI) in (**b**). **P*<0.05. Data are plotted showing the mean and standard deviation in (**a-c**). Source data are provided as a Source Data file.

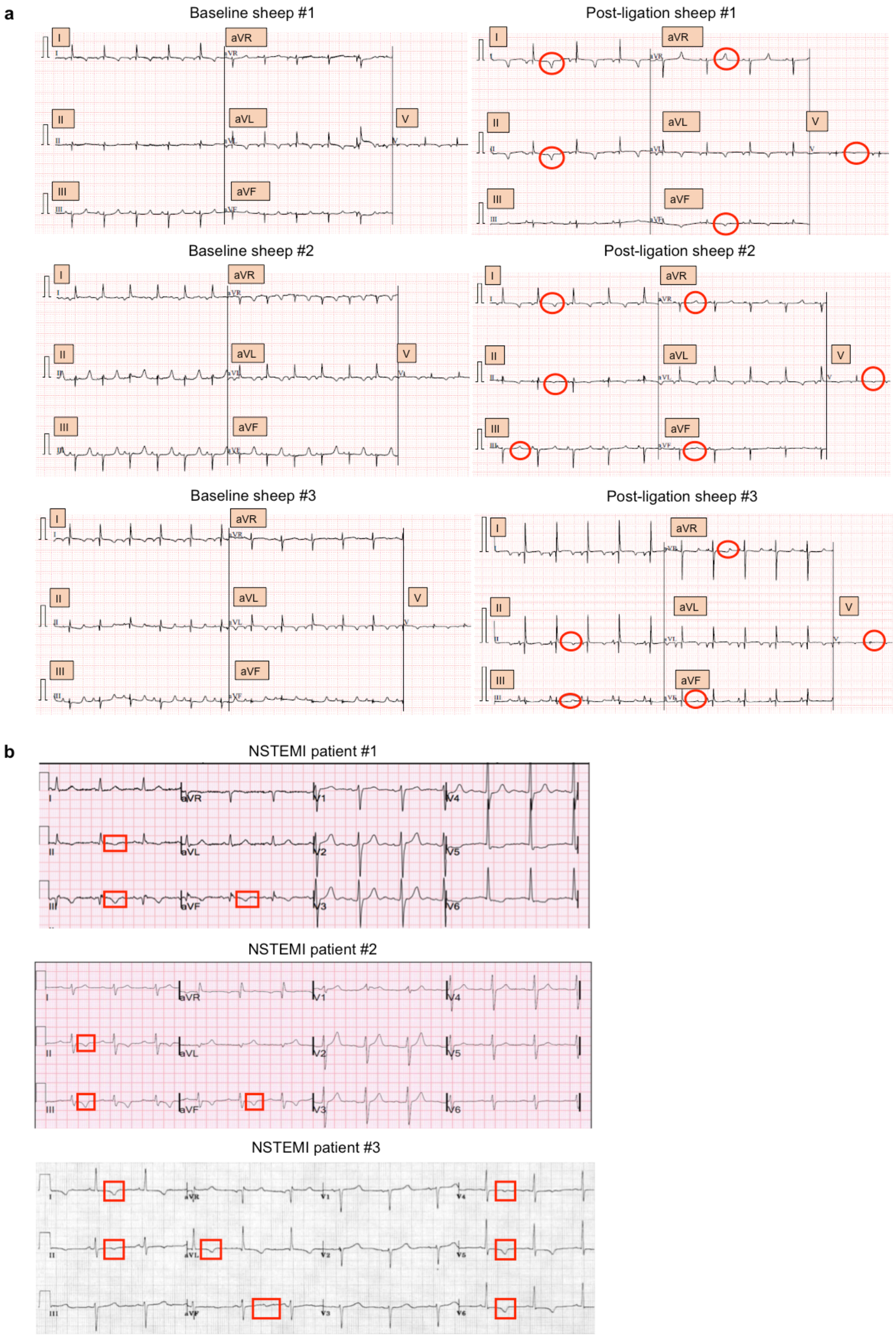
a
Baseline sheep #1
I
aVR
II
aVL
V
III
aVF
Post-ligation sheep #1
I
aVR
II
aVL
V
III
aVF
Baseline sheep #2
I
aVR
II
aVL
V
III
aVF
Post-ligation sheep #2
I
aVR
II
aVL
V
III
aVF
Baseline sheep #3
I
aVR
II
aVL
V
III
aVF
Post-ligation sheep #3
I
aVR
II
aVL
V
III
aVF
b
NSTEMI patient #1
I
aVR
V1
V4
II
aVL
V2
V5
III
aVF
V3
V6
NSTEMI patient #2
I
aVR
V1
V4
II
aVL
V2
V5
III
aVF
V3
V6
NSTEMI patient #3

Supplementary Figure 3 | Resemblance of NSTEMI model to clinical cases

a, ECG tracings before and after the induction of an NSTEMI type infarction in sheep. Baseline: ECG tracing with no significant changes in QRS, ST segments or T waves. Post-ligation changes (circled in red) in sheep #1: T wave inversion in leads I, II and aVF, accentuated peaking of T waves in aVR, with flattening of the T wave in leads III and V. Post-ligation changes (circled in red) in sheep #2: T wave inversion in leads I, II and eversion of T wave in aVR, with flattening of the T waves in leads III, aVF and V. Post-ligation changes (circled in red) in sheep #3: T wave inversion in leads II, III and eversion of T wave in aVR, with flattening of the T waves in leads aVF and V. **b**, Clinical ECG tracings in three different patients diagnosed with NSTEMI. Top, patient #1, 59-year-old complaining of persistent angina for two hours: T wave inversion with ST depression (framed in red) in leads II, III and aVF. Middle, patient #2, 67-year-old presenting with prolonged chest pain of sudden onset: T wave inversion with ST depression (framed in red) in the inferior leads II, III and aVF. Bottom, patient #3, 72-year-old with acute onset of prolonged chest pain: Widespread T wave inversion (framed in red) in the septal and inferolateral leads.

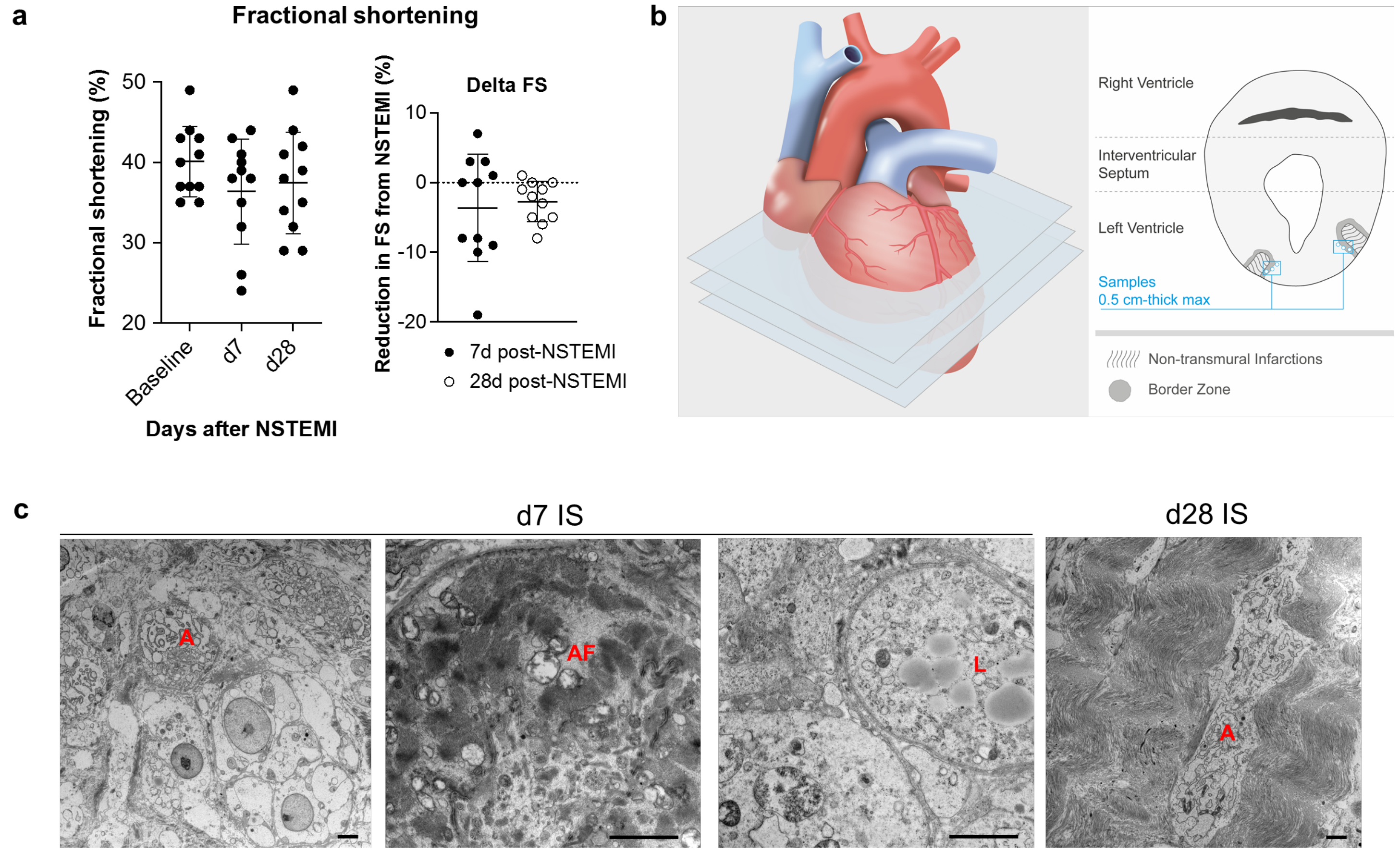

Supplementary Figure 4 | Functional and histological evaluation of the NSTEMI model

a, Left, fractional shortening (FS) percentage at baseline, on d7 and d28 post-NSTEMI. Right, reduction in FS (delta) 7 and 28 days post-surgery. *n*=11 animals. **b**, Schematics of tissue harvesting from explanted hearts. After perfusion with PBS to remove the excess of blood, each heart was cross-sectioned in slices with a thickness of 1 cm from the atrium to the apex. The whitish colour within samples identified clear infarcted areas. Samples with a maximum size of 0.5 cm in every direction were taken from the ischaemic core, the border and the remote regions. **c**, Representative TEM micrographs on d7 post-NSTEMI (left and centre): Apoptotic bodies (A), autophagosomes (AF) and lipid droplets (L) are widely spread in ischaemic regions previously populated by intact cardiomyocytes. Representative TEM micrographs at 28 days post-NSTEMI (right): Wavy-oriented typical collagen-like deposition replaced cardiomyocytes. *n*=5 animals per group, scale bar = 2 µm. One-way ANOVA with Tukey's multiple comparisons test in (**a**), and two-tailed Wilcoxon test in (**b**). Data are plotted showing the mean and standard deviation in (**a**). Source data are provided as a Source Data file.

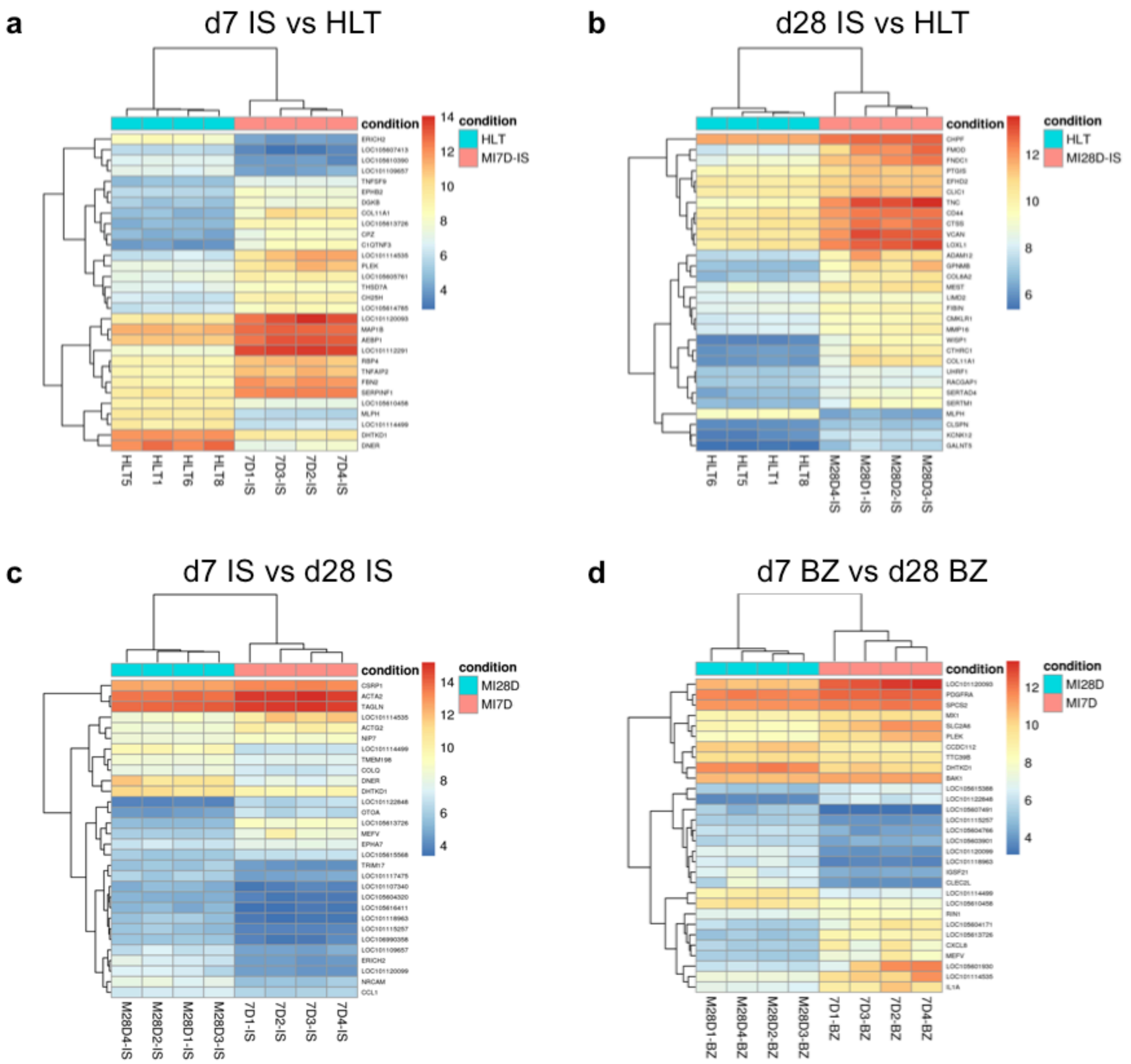

Supplementary Figure 5 | Transcriptomics in ischaemic core following NSTEMI

a,**b** Bi-clustering heat map listing the top 30 genes that are called differentially expressed (DEG) sorted by lowest p-values (P<0.05) detected in the ischaemic core on d7 (**a**) and d28 (**b**) post-NSTEMI compared with healthy (HLT) myocardial ventricular tissue from RNA-sequencing data. **c**,**d** Bi-clustering heat map of DEG between d7 and d28 post-NSTEMI ischaemic core (IS) (**c**) and border zone (BZ) samples (**d**). Difference in mRNA expression between the conditions is indicated as $\log_2$(fold change). Analysis was performed using DESeq2 package and statistical significance assessed by two-sided Wald's test using Benjamini-Hochberg method. Adjusted-P<0.05 was set to identify differences. n=4 animals per group.

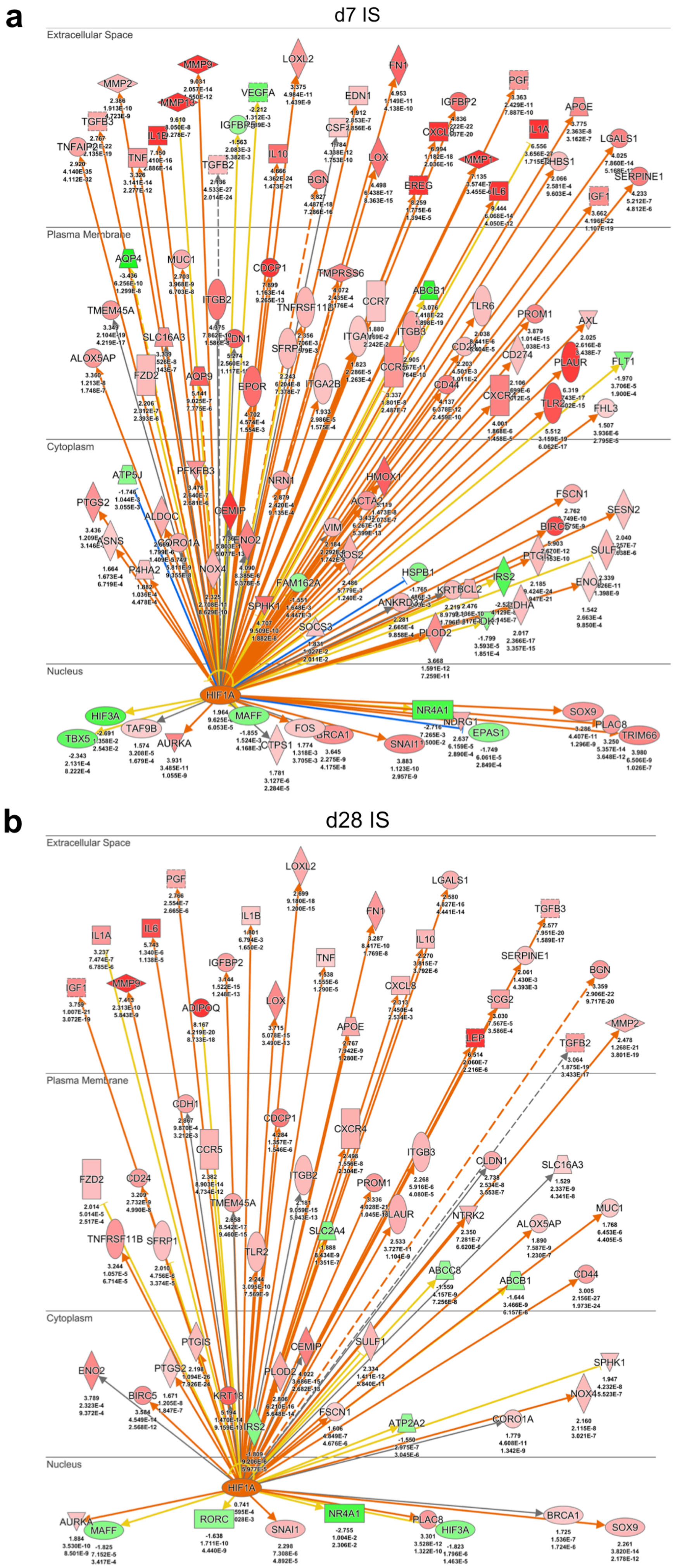
a
d7 IS
Extracellular Space
Plasma Membrane
Cytoplasm
Nucleus
MMP9
LOXL2
FN1
MMP2
MMP13
VEGFA
EDN1
PGF
IGFBP2
APOE
TGFB3
IGFBP5
CSF
CXCL8
IL1A
LGALS1
TNFAIP2
IL1B
TNF
IL10
TGFB2
LOX
BGN
EREG
MMP1
THBS1
SERPINE1
IL6
IGF1
AQP4
MUC1
CDCP1
TMPRSS6
ABCB1
ITGB2
TNFRSF11B
CCR7
TLR6
PROM1
AXL
TMEM45A
SLC16A3
ITGA
ITGB3
ALOX5AP
SFRP
FZD2
AQP9
EPOR
ITGA2B
CCR5
CD44
CD274
PLAUR
FLT1
CXCR
TLR2
FHL3
ATP5J
PFKFB3
NRN1
HMOX1
FSCN1
SESN2
PTGS2
ALDOC
CEMIP
ACTA2
BIRC5
ASNS
CORO1A
ENO2
VIM
PTG
SULF
P4HA2
NOX4
HSPB1
IRS2
FAM162A
KRT
BCL2
ENO
SPHK1
ANKRD
SOCS3
PLOD2
DHA
HIF1A
HIF3A
MAFF
NR4A1
SOX9
TAF9B
NDRG1
PLAC8
TBX5
AURKA
FOS
EPAS1
TRIM66
CTPS1
BRCA1
SNAI1
b
d28 IS
Extracellular Space
Plasma Membrane
Cytoplasm
Nucleus
LOXL2
PGF
LGALS1
IL1B
FN1
TGFB3
IL1A
IL6
IL10
TNF
SERPINE1
IGFBP2
BGN
IGF1
MMP9
CXCL8
SCG2
ADIPOQ
LOX
APOE
MMP2
LEP
TGFB2
CDH1
CDCP1
CXCR4
CCR5
ITGB3
CLDN1
SLC16A3
FZD2
CD24
ITGB2
PROM1
TMEM45A
PLAUR
NTRK2
MUC1
ALOX5AP
TNFRSF11B
SFRP1
TLR2
SLC2A4
ABCC8
ABCB1
CD44
PTGIS
CEMIP
SULF1
SPHK1
ENO2
PTGS2
PLOD2
BIRC5
KRT18
NOX4
IRS2
FSCN1
ATP2A2
CORO1A
HIF1A
AURKA
MAFF
RORC
SNAI1
NR4A1
PLAC8
HIF3A
BRCA1
SOX9

Supplementary Figure 6 | HIF1α Causal Network Analysis (IPA®) in ischaemic core post-NSTEMI

a,**b** HIF1α is a key regulator in NSTEMI on d7 (**a**) and d28 (**b**) post-NSTEMI. Shades of red and green indicate the degree of upregulation and downregulation, respectively. The activation of the downstream node is orange-coded, inhibition is blue-coded. If the findings underlying the relationship are inconsistent with the state of the downstream node yellow is used, and grey if there is no predicted effect. Pointed arrowheads indicate that the downstream node is expected to be activated, while blunt arrowheads indicate it is expected to be inhibited. $\log_2$(fold change), *P*, and adjusted-*P* from RNA-sequencing data are indicated below the gene names. *n*=4 animals per group.

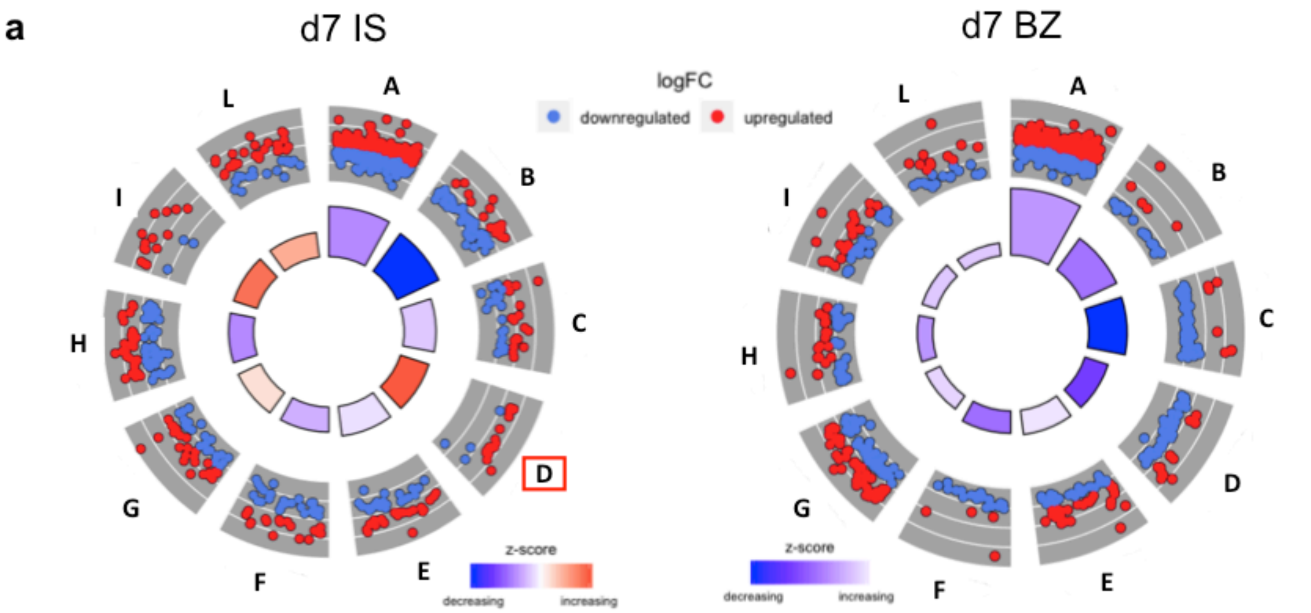

ID	Description
A	Metabolic pathways
B	Ubiquitin mediated proteolysis
C	Glycerophospholipid metabolism
D	N-Glycan biosynthesis
E	Neurotrophin signaling pathway
F	FoxO signaling pathway
G	Hippo signaling pathway
H	MAPK signaling pathway
I	DNA replication
L	Cell cycle

ID	Description
A	Metabolic pathways
B	Citrate cycle (TCA cycle)
C	Oxidative phosphorylation
D	Ubiquitin mediated proteolysis
E	FoxO signaling pathway
F	Pyruvate metabolism
G	MAPK signaling pathway
H	Insulin signaling pathway
I	AMPK signaling pathway
L	Glycolysis / Gluconeogenesis

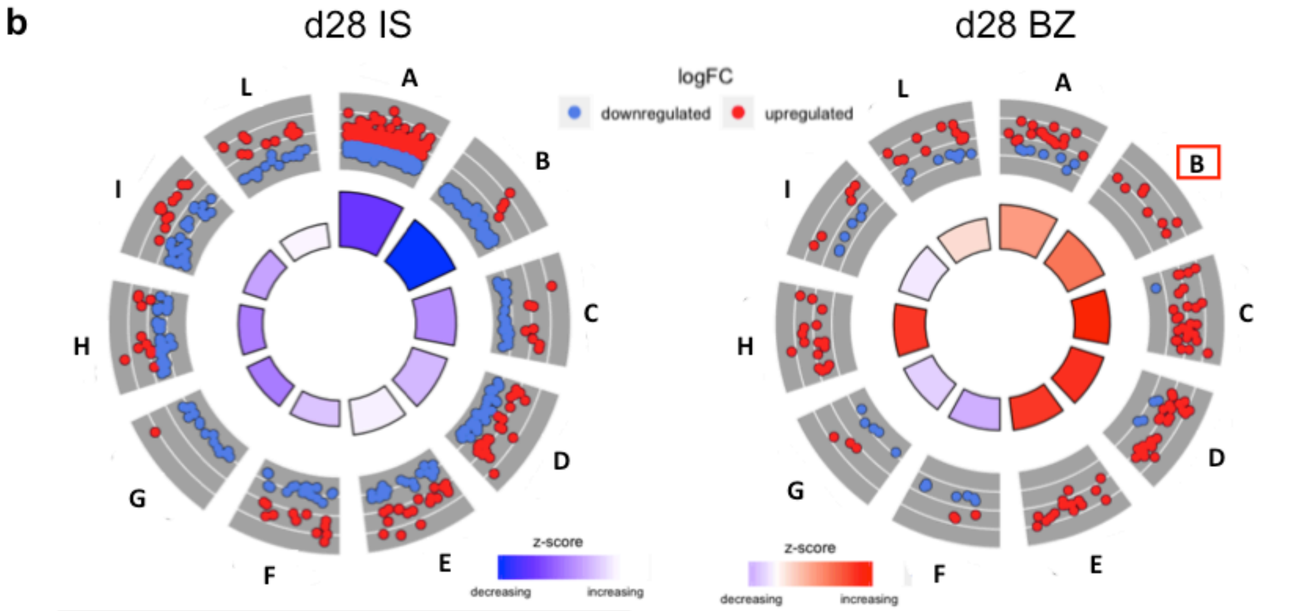

ID	Description
A	Metabolic pathways
B	Oxidative phosphorylation
C	Cardiac muscle contraction
D	Adrenergic signaling in cardiomyocytes
E	Glycerophospholipid metabolism
F	Dilated cardiomyopathy
G	Citrate cycle (TCA cycle)
H	Insulin signaling pathway
I	Glucagon signaling pathway
L	Arrhythmogenic RV cardiomyopathy

ID	Description
A	Cell cycle
B	Glycosaminoglycan biosynthesis
C	Chemokine signaling pathway
D	Focal adhesion
E	Fc gamma R-mediated phagocytosis
F	Fatty acid degradation
G	Proponoate metabolism
H	ECM-receptor interaction
I	Fatty acid metabolism
L	FoxO signaling pathway

Supplementary Figure 7 | Functional annotation analysis on transcriptomics

a,**b** Functional annotation analysis using DAVID software of the DEG in ischaemic core and border zone following NSTEMI. Gene-annotation enrichment analysis (KEGG_PATHWAY) of the set of DEG (adjusted-$P<0.05$) is displayed using the GOCircle plot in which the inner ring is a bar plot where the height of the bar indicates the significance of the term -Log(adjusted-*P*), and the colour corresponds to the z-score. Red dots represent up-regulated genes and blue dots indicate down-regulated genes. **a**, N-glycan biosynthesis (framed in red) has one of the highest z-scores in the ischaemic core (IS) on d7 post-surgery. **b**, Glycosaminoglycan biosynthesis (framed in red) is one of the top biological categories showing a highly activated z-score in the border zone (BZ) on d28 post-surgery. *n*=4 animals per group.

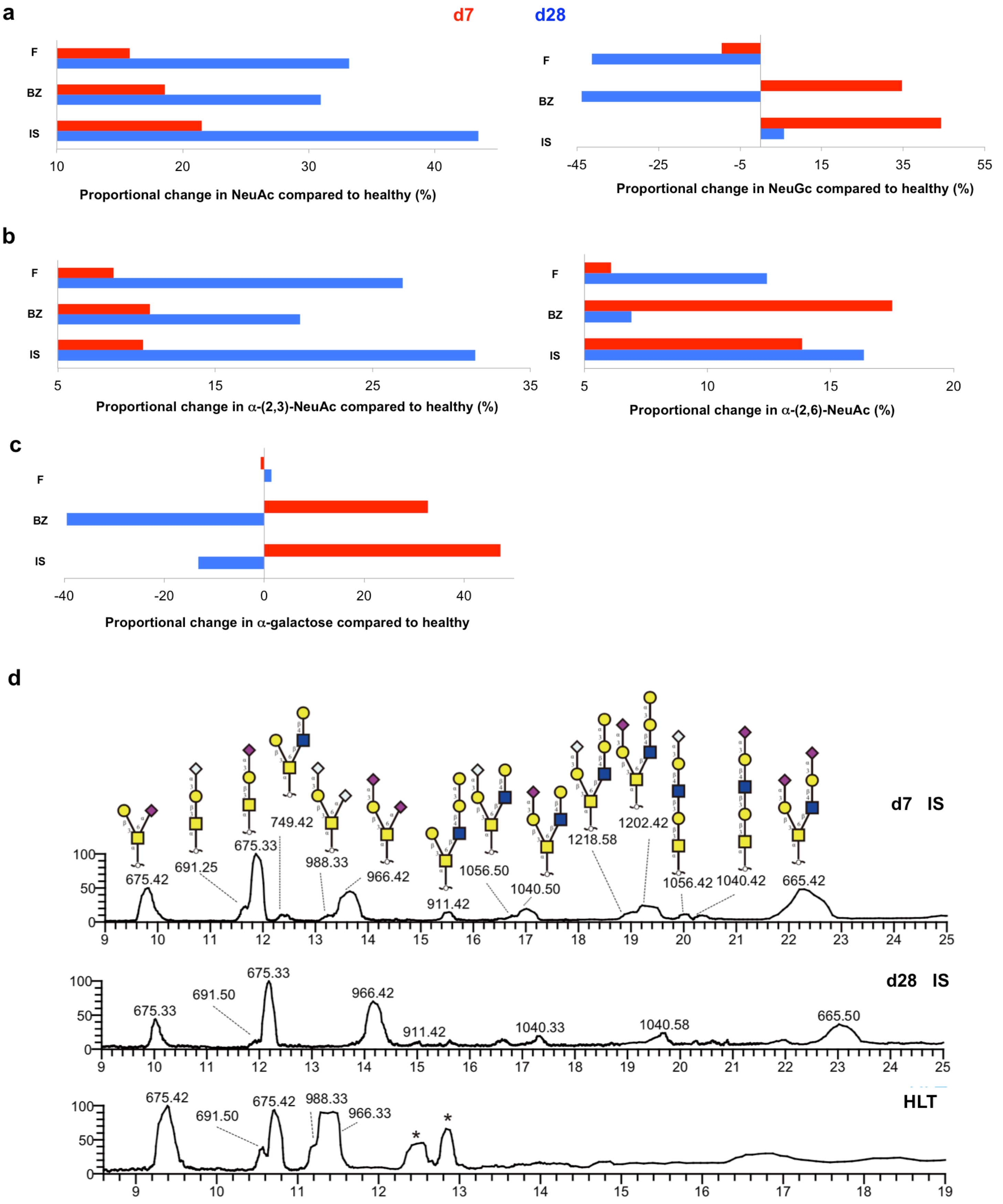

Supplementary Figure 8 | Glycomics analysis of infarcted hearts following NSTEMI

Proportional changes in N-linked glycans in the ischaemic core, border and remote regions compared to healthy. **a**,**b** Amount of N-linked glycans expressing either NeuAc or NeuGc sialic acid (**a**) and α(2,3)- or

α(2,6)- linkage type (**b**) across the different regions on d7 and d28 post-NSTEMI. **c**, Amount of terminal α-gal in the infarcted regions. **d**, Extracted ion chromatography (EIC) showing O-linked glycans mainly detected in the cellular membrane protein extracts from the ischaemic core on d7 and d28 post-surgery compared with healthy (HLT) myocardial tissue. Regions of infarcted hearts are labelled as follows: IS = ischaemic core, BZ = border zone, F = remote zone from the infarct. Data are representative of two independent experiments. Each analysed sample was a pool of samples coming from three individuals (*n*=3 animals per group). Source data are provided as a Source Data file.

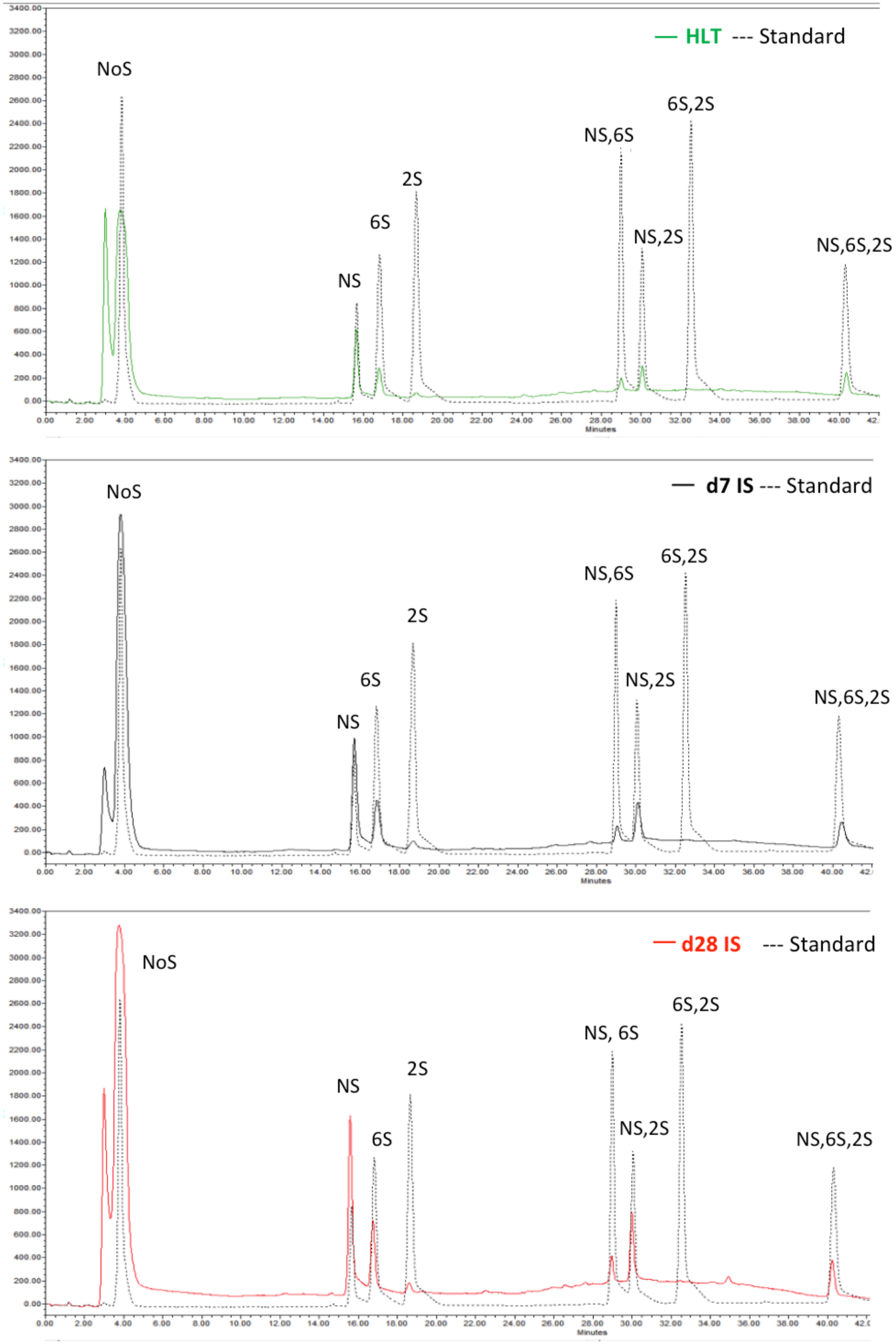

HLT --- Standard
NoS
NS
6S
2S
NS,6S
NS,2S
6S,2S
NS,6S,2S
Minutes
d7 IS --- Standard
NoS
NS
6S
2S
NS,6S
NS,2S
6S,2S
NS,6S,2S
Minutes
d28 IS --- Standard
NoS
NS
6S
2S
NS, 6S
NS,2S
6S,2S
NS,6S,2S
Minutes

Supplementary Figure 9 | HS sulfation pattern following NSTEMI

Representative HPLC chromatograms showing the sulfation profile of heparan sulfate (HS) extracted from healthy myocardial tissue (top), and the ischaemic region on d7 (middle) and d28 (bottom) post-NSTEMI. Each profile has been integrated by comparison to an external standard mix (dotted line in grey in all chromatograms) composed of unsulfated disaccharide (NoS), monosulfated disaccharides with a different position of sulfation (NS; 6S; 2S), disulfated disaccharides (NS, 6S; NS,2S; 6S, 2S) and trisulfated disaccharide (NS, 6S, 2S). *n*=5 HLT, *n*=4 d7 and *n*=5 d28 post-NSTEMI.

Abbreviations

ACN: Acetonitrile

AHA: American Heart Association

AUC: Area Under the Curve

BSA: Bovine Serum Albumin

BZ: Border Zone

CID: Collision-Induced Dissociation

CS: Chondroitin Sulfate

cTn: Cardiac Troponin

DAVID: Database for Annotation, Visualization, and Integrated Discovery

DEG: Differential Expressed Genes

DMMB: Dimethylmethylene Blue

ECG: Electrocardiogram

ECM: Extracellular Matrix

EDD: left ventricular End Diastolic Diameter

EDTA: Ethylenediaminetetraacetic Acid

EF: Ejection Fraction

ESD: left ventricular End Systolic Diameter

F: Remote region

FA: Formic Acid

FBS: Foetal Bovine Serum

FDR: False Discovery Rate

FS: Fractional Shortening

GAGs: Glycosaminoglycans

GO: Gene Ontology

HPLC: High Performance Liquid Chromatography

HS: Heparan Sulfate

IL: Interleukin

IPA: Ingenuity Pathway Analysis

IS: Ischaemic core

KEGG: Kyoto Encyclopedia of Genes and Genomes

LAD: Left Anterior Descending coronary artery

MI: Myocardial Infarction

MMP: Matrix Metalloproteinase

MWCO: Molecular Weight Cut-Off

NeuAc: Neuraminic Acid

NeuGc: N-Glycolylneuraminic Acid

NSTEMI: Non-ST-segment Elevation Myocardial Infarction

O/N: Overnight

PB: Phosphate Buffer

PBS: Phosphate Buffer Saline

PFA: Paraformaldehyde

LC-ESI-MS/MS: Liquid Chromatography-Electrospray Ionization-tandem Mass Spectrometry

PK: Proteinase K

RT: Room Temperature

sGAG: Sulfated Glycosaminoglycan

SIGLEC: Sialic acid-binding Immunoglobulin-type Lectin

SMA: Smooth Muscle Actin

STEMI: ST-segment Elevation Myocardial Infarction

TBS: Tris-Buffered Saline

TEAB: Triethylammonium Bicarbonate

TEM: Transmission Electron Microscopy

TFA: Trifluoroacetic Acid

TKO: Triple Knock-out

TNF: Tumour Necrosis Factor

TTE: Transthoracic Echocardiography

UHPLC: Ultra-High Performance Liquid Chromatography

VEGF: Vascular Endothelial Growth Factor

WMI: Wall Motion Index

α-Gal: α-Galactose
