# Supplementary material for: Reproducing extracellular matrix adverse remodelling of non-ST myocardial infarction in a large animal model
Source: Nat Commun. 2023 Feb 22;14:995. doi: 10.1038/s41467-023-36350-1 (PMC9945840; doi:10.1038/s41467-023-36350-1)
Supplement: Supplementary file 3 — Description of Additional Supplementary Files [file 41467_2023_36350_MOESM3_ESM.pdf]

Description of Additional Supplementary Files

Reproducing extracellular matrix adverse remodelling of non-ST myocardial infarction in a large animal model

Paolo Contessotto, Renza Spelat *et al*.

Supplementary Data

Supplementary Data 1:
Differentially expressed genes (DEG) list of ischemic core region on day 7 (d7) vs day 28 (d28) post-NSTEMI

Supplementary Data 2:
DEG list of border zone region on d7 vs d28 post-NSTEMI

Supplementary Data 3:
DEG list of remote zone region on d7 vs d28 post-NSTEMI

Supplementary Data 4:
DEG list of ischemic core region on d7 post-NSTEMI vs healthy

Supplementary Data 5:
DEG list of ischemic core region on d28 post-NSTEMI vs healthy

Supplementary Data 6:
Canonical Pathway Analysis of ischemic core region DEG on d7 and d28 by IPA®

Supplementary Data 7:
Biological Functions Analysis of ischemic core region DEG on d7 and d28 by IPA®

Supplementary Data 8:
Molecular targets associated with HIF1α on d7 ischemic core obtained by IPA® from DEG data

Supplementary Data 9:
Molecular targets associated with HIF1α on d28 ischemic core obtained by IPA® from DEG data

Supplementary Data 10:
Upstream Regulator Analysis of ischemic core on d28 by IPA® from proteomic data

Supplementary Data 11:
List of proteins detected by nLC-ESI MS/MS label-free proteomic analysis (false-positive identification rate FDR ≤1%) in ischemic core (IS), border zone (BZ), remote zone (F) on d7 and d28 post-NSTEMI and healthy (HLT) samples

Supplementary Data 12:
Gene Ontology Enrichment Analysis of ischemic core region DEG on d7 post-NSTEMI

Supplementary Data 13:
Gene Ontology Enrichment Analysis of border zone region DEG on d7 post-NSTEMI

Supplementary Data 14:
Gene Ontology Enrichment Analysis of ischemic core region DEG on d28 post-NSTEMI

Supplementary Data 15:
Gene Ontology Enrichment Analysis of border zone region DEG on d28 post-NSTEMI

Supplementary Data 16:
List of N- and O-glycans detected by LC-ESI MS/MS glycomic analysis in ischemic core (IS), border zone (BZ), remote zone (F) on d7 and d28 post-NSTEMI and healthy (HLT) samples
